# New Advertisements

**Published:** 1886-05-20

**Authors:** 


### EDITORIALS AND MISCELLANEOUS.

### NEW ADVERTISEMENTS.

CHARLES TRUAX & CO.—We invite attention to the advertisement, in this number, of the above house, who deal in Elastic Stockings, Supporters, Bed-pans, Douche Apparatus, etc. We have one of their bed-pans and douche, and think them very convenient and useful.

MALTINE —This preparation, with its various combinations with the phosphates, iron, quinia, cod-liver oil, strychnia, etc., has been found to fill numerous conditions in practice. See the advertisement of this excellent article by the Maltine Manufacturing Company as found in this number of our journal. The house has a high reputation for reliability, while their goods are of a superior order.

ISAAC PHILLIPS, the Surgical Instrument dealer, was present at the late meeting of the Georgia Medical Association, with a large and beautiful display of surgical Instruments. Mr. Phillips is a polite and accommodating gentleman, and an enterprizing business man. He has an advertisement in this number of the RECORD.

PARKE, DAVIS & CO., with their usual enterprise, had on exhibition at the Association samples of their beautiful and popular preparations. They have an advertisement in this number of the RECORD.

